# Patient-derived conditionally reprogrammed cells maintain intra-tumor genetic heterogeneity

**DOI:** 10.1038/s41598-018-22427-1

**Published:** 2018-03-06

**Authors:** Bruna R. S. Correa, Joanna Hu, Luiz O. F. Penalva, Richard Schlegel, David L. Rimm, Pedro A. F. Galante, Seema Agarwal

**Affiliations:** 1 Centro de Oncologia Molecular - Hospital Sírio-Libanês, São Paulo, SP 01308-060 Brazil; 2 Department of Pathology, Yale University, New Haven, CT 06510 USA; 3 Children’s Cancer Research Institute – UTHSCSA, San Antonio, TX 78229 USA; 4 Department of Pathology, Center for Cell Reprogramming, Georgetown University Medical Center, Washington, DC 20007 USA; 5 Present Address: Centre for Genomic Regulation (CRG), Barcelona, 08003 Spain

## Abstract

Preclinical *in vitro* models provide an essential tool to study cancer cell biology as well as aid in translational research, including drug target identification and drug discovery efforts. For any model to be clinically relevant, it needs to recapitulate the biology and cell heterogeneity of the primary tumor. We recently developed and described a conditional reprogramming (CR) cell technology that addresses many of these needs and avoids the deficiencies of most current cancer cell lines, which are usually clonal in origin. Here, we used the CR cell method to generate a collection of patient-derived cell cultures from non-small cell lung cancers (NSCLC). Whole exome sequencing and copy number variations are used for the first time to address the capability of CR cells to keep their tumor-derived heterogeneity. Our results indicated that these primary cultures largely maintained the molecular characteristics of the original tumors. Using a mutant-allele tumor heterogeneity (MATH) score, we showed that CR cells are able to keep and maintain most of the intra-tumoral heterogeneity, suggesting oligoclonality of these cultures. CR cultures therefore represent a pre-clinical lung cancer model for future basic and translational studies.

## Introduction

Intra-tumor heterogeneity (ITH), defined by the coexistence of genetically distinct sub-clonal populations of cells within the same tumor, is the most relevant feature of all cancers and defines the response to a given therapy, cellular dissemination and progression of primary tumor^1–4^. Although we have been aware of ITH since the early 1980’s via cytogenetic studies^5^, only recently has its complexity and implications been appreciated, thanks to the advent of high throughput approaches such as next generation sequencing (NGS)^1,2,6^. Conventional cell line models failed to capture this important aspect of tumors as they are mostly clonal in nature. Patient derived tumor xenografts (PDXs) are able to capture the intra-tumor heterogeneity^7–10^, but the success rate of establishing these models is not very high and it is not very cost-effective, especially for drug discovery studies^8,9,11–14^.

Here, we assess the capability of conditional reprogramming (CR)^15,16^ of cells to keep their tumor derived heterogeneity and morphological features. We established 10 individual primary cell lung cancer cultures directly from patient’s tissue samples using conditionally reprogram (CR) technology. Whole exome sequencing (WES) and copy number variations (CNVs) were used to assess the level of ITH in cell cultures when compared with primary tumor and normal tissue materials from each patient. Our results indicate that patient-derived cell model system using CR technology is able to capture intra-tumor heterogeneity in addition to maintaining the morphological features.

## Results

### Genomic Intra-tumor heterogeneity of primary tumors is maintained in CR cells

**Figure 1:**
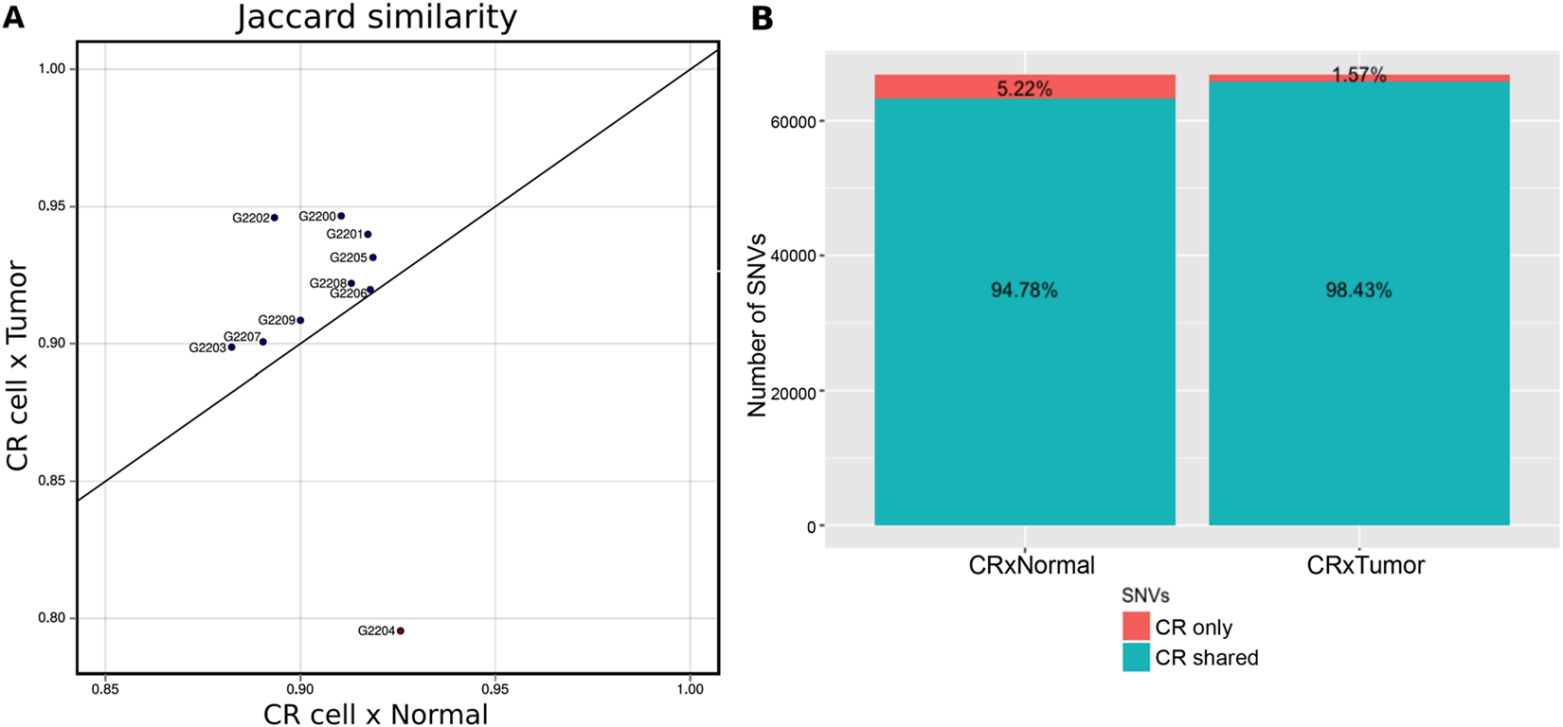
Tumor conditionally reprogrammed cells are more similar to the tumor of origin rather than the corresponding normal tissue. (**A**) Jaccard similarity plot show that tumor conditionally reprogrammed (CR) cultures are more similar to their corresponding tumor tissue rather than normal. (**B**) Bar plot showing the precentage of single nucleotide variations (SNVs) shared by CR cells x primary tumor and CR cells x normal tissues.

CR Lung cancer cultures were established directly from tissue samples from ten individual patients (Table 1) who were diagnosed with non-small cell lung cancer. These cultures maintained the morphological features of the tumor of origin (Supplemental Fig. S1). In order to address the capability of CR cells to maintain their tumor-derived heterogeneity, we carried out exome sequencing and single nucleotide variation calling from normal tissue, primary tumor and CR cells. To test whether the cancer CR cells shared the genomic features with primary tumor, we used a Jaccard Index that is commonly used for comparing the similarity and diversity of sample sets^17^. Based on the Jaccard similarity (1 – Jaccard distance), we found that all CR cells (exception to G2204) are located in the upper quadrant suggesting that they are more similar in term of their SNVs to tumors than to normal (Fig. 1A). In total, CR cells share 98.43% of their SNVs with primary tumors, while only 94.78% of CR cells SNVs are shared with normal tissues (Fig. 1B). These data also indicate that all tumor CR cell cultures are contaminated with normal cells present in the patient’s tissue samples, the CR technology does not differentiate between the growth of both normal and tumor cells.

**Table 1:** Summary of patient’s clinical information.

Specimen ID	Tumor Type	Age	Stage	Gender	Diagnosis
G2200	Carcinoid	33	IB	F	Primary
G2201	SCC*	72	IIA	M	Primary
G2202	ADCA**	78	IA	F	Primary
G2203	ADCA	46	IV	M	met from colorectal cancer
G2204	SCC	66	IIA	M	Primary
G2205	ADCA	75	IA	M	Primary
G2206	ADCA	76	IB	M	Primary
G2207	SCC	81	IB	M	Primary
G2208	Large cell neuroendocrine	58	IA	F	Primary
G2209	Mucinous ADCA	57	IB	M	Primary

*SCC: Squamous cell carcinoma, **ADCA: Adenocarcinoma.

**Figure 2:**
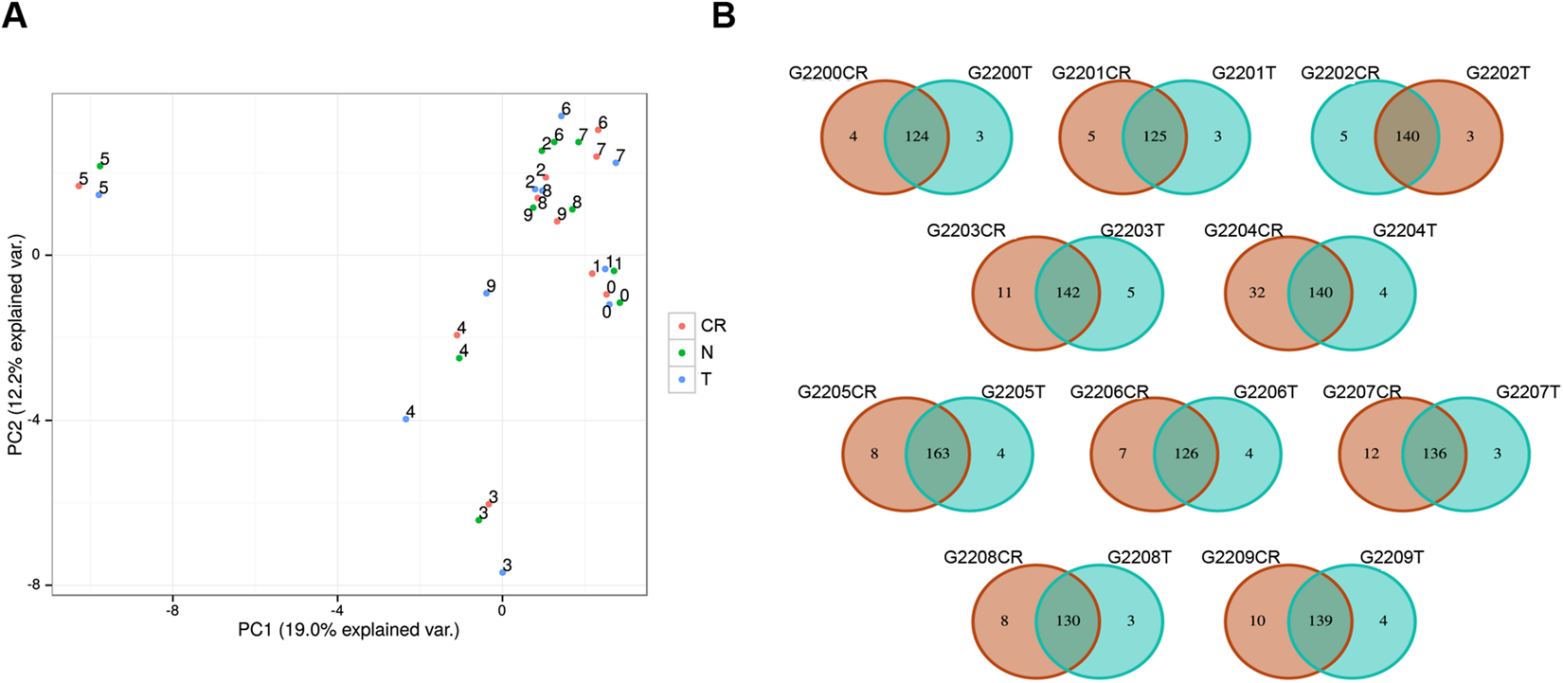
The genetic relationship between conditionally reprogrammed cells, primary tumor and normal cells show good correlation. (**A**) Principal component analysis reveal that SNVs for each patient’s normal, tumor and conditonally reprogrammed (CR) cells cluster together indicating that CR cells maintained the genetic features of the patient. (**B**) Venn diagram of SNVs in cancer genes for each CR cell culture (CR) compared to the corresponding primary tumor (T) show highly similar mutation profile.

Do CR cells have a SNV profile grouped to condition or origin? In order to answer this question, we evaluated the individual profile of SNVs in each of our 10 CR cells by performing a principal component analysis (PCA) to assess the genetic distance and relatedness between populations. We analyzed all SNVs and also only those SNVs specifically present in cancer genes^6^. We selected these cancer-specific SNVs panel because several malignant cells arise as a result of somatic changes in the cancer genes. Figure 2A shows that each triplet (CR cell, primary tumor and normal tissue) is grouped, indicating that CR cells still keep the idiosyncrasy from their tissue of origin (patient). The genetic relationship between CR cells and primary tumor for cancer genes shows a good correlation (>90%), with the exception of G2204, presented as the Venn diagram in Fig. 2B. The differences between tumor of origin and CR cells can be due to the failure of CR culture conditions to propagate some of the clones from the primary tumor, thus showing SNVs in tumor but not in the CR cells. Secondly, it is possible that some clonal populations were present in the original tumor in such a low level that it escaped the detection, but grew out under CR culture conditions, hence the unique SNVs in the CR culture, but not present in the primary tumor. Third scenario can be the highly heterogeneous nature of a given tumor, thus the tumor piece used for sequencing likely will have different mutational spectrum compared to the one that was used to establish CR cultures. Fourth is the combination of all above possibilities. Fifth, there is a possibility that novel mutations might arise under CR culture conditions, but this seems unlikely since all different clones in 10 individual cases are entirely different from each other, which would not be the likely outcome of culture-induced mutations.

**Figure 3:**
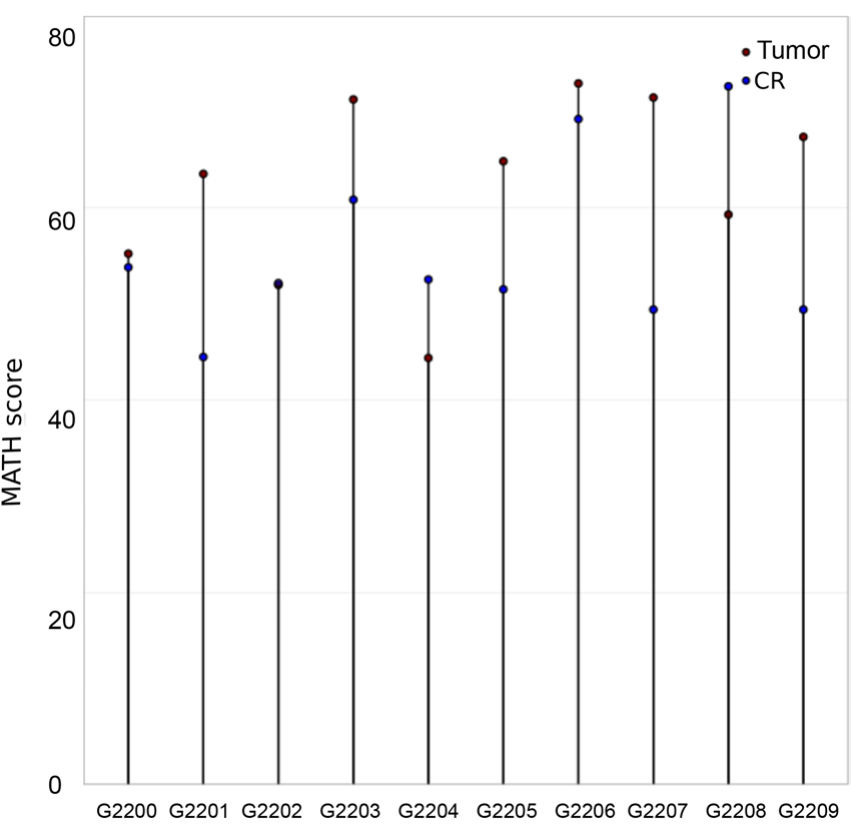
Conditionally reprogrammed cells maintain the intra-tumor heterogeneity of the primary tumor. Mutant-allele tumor heterogeneity (MATH) scoring for primary tumor (red circle) and the corresponding tumor conditionally reprgrammed (CR cells, blue circle) excluding SNVs in corresponding normal samples show a high degree of overlap.

Next, in order to evaluate if CR still keep the heterogeneity (ITH) from their respective primary tumors, we used the mutant-allele tumor heterogeneity (MATH) score. MATH is a novel, un-biased, quantitative method developed to measure the intra tumoral heterogeneity (ITH)^18^ based upon the number and frequency of SNVs obtained through next generation exome sequencing. This method not only allows the direct identification and enumeration of tumor cell subpopulations, but it also quantifies and compares sample heterogeneity levels. It is expected to have little influence of CNV in MATH score, which is determined as the ratio of the width to the center of the distribution of mutant allele fractions (MAFs) for tumor-specific point mutations. We observed that all primary tumor samples (except G2204 and G2208) present higher MATH scores than their respective CR cells (Fig. 3), but all CR cells kept intra-tumor heterogeneity. Interestingly, three primary tumors and their respective CR cells (G2200, G2202 and G2206), presented a very similar MATH score, suggesting that CR cells capture almost all of tumor ITH. On the other hand, G2203 and G2205 CR cells presented a smaller MATH score than their primary tumor, indicating that CR cells were not able to capture all ITH from primary tumors although they are not clonal like standard cell lines. Moreover, G2204 and G2208 had an unexpected higher MATH score than its corresponding tumor tissue. These differences in MATH score among cell line vs corresponding primary tissue are not very surprising given the heterogeneous nature of the tumor. It is likely that the region of tissue sample that was used to establish CR culture may be different from the one that was used for sequencing. As discussed above these differences can be explained in several ways. However, it is striking that CR cultures are largely maintaining the heterogeneity of the tumor of origin at least by >90%.

### Copy number variation of originating tumor is largely preserved in cell culture

**Figure 4:**
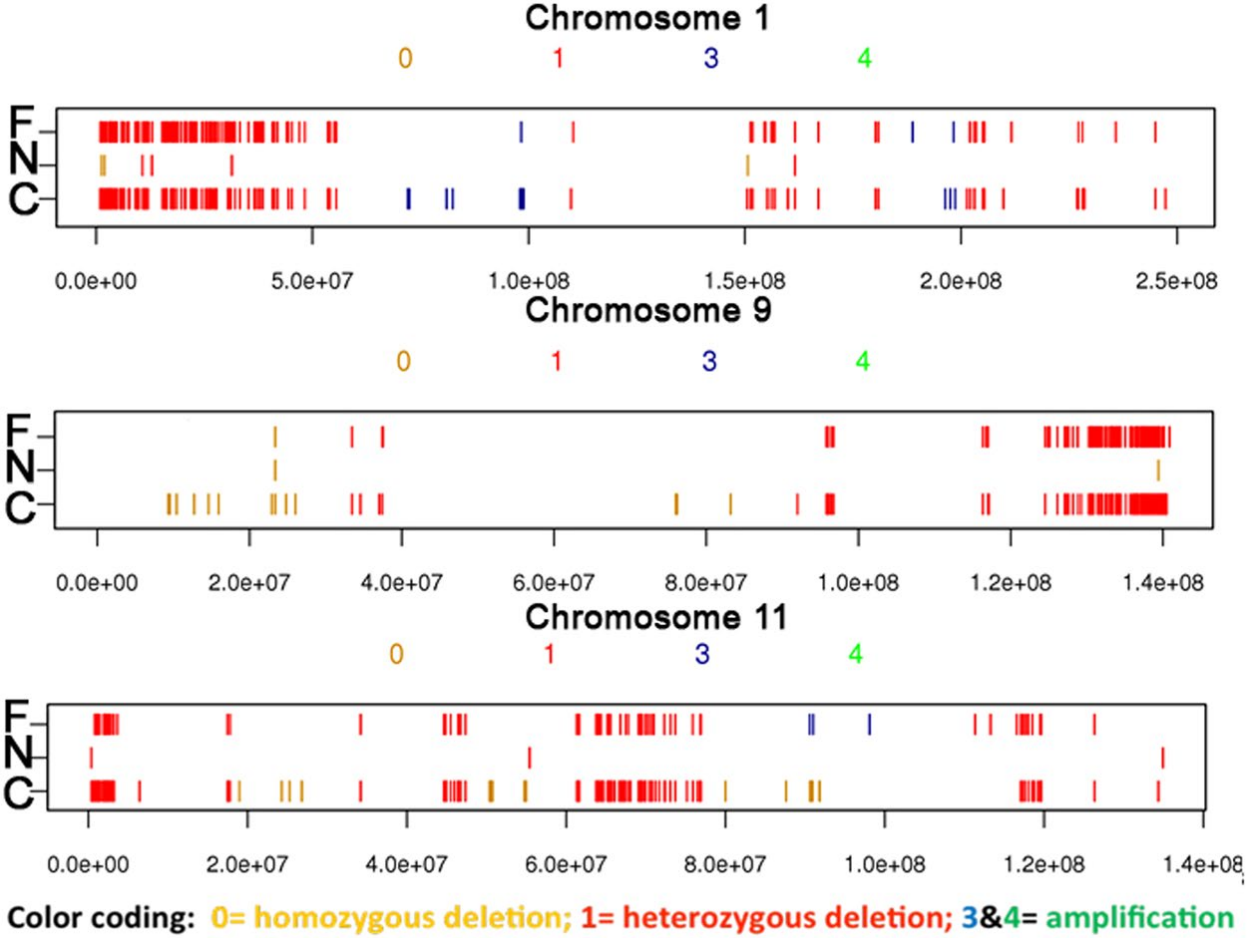
Conditionally reprogrammed cells show similar copy number variation profile as the primary tumor. Primary tumor tissue and tumor condtionally reprogrammed (CR) cells from patient G2202 show overlapping copy number variation (CNV) profile while adjacent normal CR cells show a very limited CNV profile as shown by high-resolution PennCNV plots for three individual chromosomes.

Copy number variation (CNV) is an important parameter for the intra-tumor heterogeneity^19,20^. In order to assess this type of variation in our data, we take sample from an adenocarcinoma (ADCA) patient (G2202) and compared the CNV profile of the primary tumor, adjacent normal CR culture and tumor CR culture using PennCNV, an integrated Markov model for copy number variation analysis from whole-genome SNP genotyping data at a high kilobase-resolution for each chromosome^21,22^. As expected, a normal CR culture did not show a high level of CNV compared to the tumor samples. The CNV profile of tumor CR culture largely overlapped with the primary tumor as shown in Fig. 4 and in Supplemental Fig. 2S, suggesting that this tumor CR culture (G2202) represents the primary tumor diversity for the CNV profile.

## Discussion

Intra-tumor heterogeneity is one of the primary reasons for *in vivo* drug resistance seen among cancer patients irrespective of whether it is *de novo* or acquired resistance. Drug resistance has been studied in two ways either involving conventional cell lines that are sensitive or resistant to the drugs or the sensitive cell lines were made resistant to a given drug by exposing it for a long-term. This approach even though resulted in drug resistant cell models and have provided valuable information, but given their clonal cell properties lacked the translational utility. Another approach that is rapidly gaining track is the genetic sequencing analysis of sensitive and resistant tumor tissue materials obtained before and after drug treatment often in the neoadjuvant setting. This did prove to be very informative to identify the novel genetic alterations in the resistant tumor cells and led to hypothesis-driven discovery, but due to lack of cell model system from the same patient made it impossible to test the role of these novel genetic alterations in drug resistance. Recently, a patient-derived CR model system has been reported for Recurrent Respiratory Papillomatosis (RRP)^23^, neuroendocrine^24^, prostate^25^, and lung cancer^26^ without addressing whether these patient-derived models were heterogeneous or not in nature? In this paper, we used the patient-derived lung cancer CR models to address the issue of ITH. Data provided in this paper clearly show that the patient-derived models are able to capture the heterogeneity of the primary tumors.

We are able to identify some novel SNVs that are not represented in the primary tumor and they are all different suggesting a likely possibility that these SNVs in reality may be present in the primary tumors, but at a very low level defying the detection limit and were able to selectively grow in the CR method. Whether these novel clonal cell populations have a role in tumor progression and metastasis is unknown. Recently^27^ it was shown that CR technology was successful in identifying low frequency high impact actionable mutations in primary breast cancer and liver metastasis patients. There were subset of clones that were propagated from primary tumors, but are known to present only in the brain metastasis and not in primary tumor. Similarly, CR cultures of liver metastasis identified several enriched mutations that were common among various cancer types irrespective of the primary site of tumor. Thus, this recent report and our study suggest that CR cultures will be useful in identifying rare subclones from primary tumors that become relevant for metastasis later and will likely be useful as metastasis-specific drug targets.

Having access to these primary patient-derived oligoclonal cell cultures is a huge leap forward for studying the temporal steps that lead to acquired drug resistance. This would provide an opportunity to better understand the evolution of cancer cells to go from sensitive to resistance. Usefulness of CR cultures to study the acquired resistance in lung cancer patients has been already reported by other groups, but these studies did not assess the full spectrum of ITH for those cultures^26,28^. In this report, we confirmed that these models can be useful not only for drug discovery and personalized medicine approach, but also to model the drug resistance and to better understand the biology of the inter-play between various clonal populations within a tumor that leads to tumor progression and metastasis.

One limitation of the CR technology is that it allows the growth of normal cells along with the tumor cells leading to mixed normal-tumor cultures. As shown in our data, all cultures suffered with about 50% of normal cell contamination. However, even then it was possible to show the maintenance of the intra-tumor heterogeneity among all 10 individual cancer cell cultures indicating the power of the CR technology when combined with exome sequencing. This does bring upfront the need for a better and quick method to procure the tumor tissue sample from patients that is ≥90% tumor cells to obtain as pure tumor cell culture as possible. Pathological evaluation of the tumor tissue sample is required before the cell culture.

Efforts have also been underway to establish tumor-associated fibroblast directly from patient’s tumor samples. If successful, then we can envision a model system that can capture the interaction of heterogeneous tumor cell populations with stromal component (fibroblast) to provide a first cancer model system with far reaching potential for both basic and translational research and will be useful for applications in clinical settings.

## Methods

All methods presented here were performed accordance with the relevant guidelines and regulations approved by Yale University.

### Patient-derived cell lines

All lung tissues were collected at Yale University medical school with the informed consent of the patient according to Yale University’s Institutional Review Board approval. All clinical information presented in Table 1 was obtained under de-identified clinical classification. Cell cultures were established using CR cell protocol^15,16^ and cells were maintained under these conditions at 37 °C with 5% CO_2_ in a humidified chamber.

### Whole Exome Sequencing (WES)

Formalin-fixed paraffin-embedded normal (lymph node) and tumor samples from each patient were used to isolate DNA using RecoverAll total nucleic acid isolation kit (Ambion, ThermoFisher, USA). DNA was isolated from CR cells using Qiagen’s DNeasy blood and tissue kit. Sequencing was done at Yale University’s Keck Center for Genomic Analysis. Briefly, the exomes were captured using Nimbelgen SeqCap EZ V2 human exome capture library and sequencing was performed on Illumina HiSeq 2000 in 75 base paired-end cycle mode. The sequences have been uploaded to ENA (https://www.ebi.ac.uk/ena) with the accession number PRJEB23030.

### Sequence mapping and filtering

We performed a multistep read mapping and filtering. First, all reads were mapped against the human reference genome (hg19/GRCh37.1) [https://genome.ucsc.edu] using BWA mem (default parameters)^29^. Second, all unmapped reads were selected and used in a new round of mapping against the same reference genome using NovoAlign (parameters: -o Softclip -e 10 -p 20,10 0.8,10 -s 5; www.novocraft.com). Next, in order to removed any potential mouse DNA contamination, all reads were mapped against the mouse reference genome (mm10/GRCm38) using BWA mem (default parameters)^29^ and we removed those reads mapped with highest matching score against the mouse genome. Then, PCR duplicates generated during library construction were removed using SAMtools rmdup^30^. Finally, only reads presenting mapping quality (Q) greater than 20 and uniquely mapped in the genome were selected.

### SNVs calling

We used SAMtools mpileup and bcftools^30^ to detect single nucleotide variations (SNVs). A minimal number of 3 reads (base quality Q > 30; Phred Scale) supporting the variant allele was required. Additionally, we selected only SNVs reported by reads mapped on both genome strands. We also required a minimal number of 3 reads covering the SNV genomic position in all the three conditions (Normal, Tumor, and CR Line) of each sample set.

### Jaccard Index

In order to quantify the similarity between pairs of conditions (CR Line vs. Normal; Tumor vs. Normal), we calculated the Jaccard Index using the R package *sets* (function *set similarity*, method “Jaccard”) [https://cran.rproject.org/web/packages/sets]. All SNVs in 125 cancer genes defined by Vogelstein *et al*.^6^ were selected to estimate the similarity between conditions.

### MATH scoring

To the ITH level estimation and enumeration of tumor cell subpopulations, we used the mutant-allele tumor heterogeneity (MATH) score. The MATH score was calculated as originally described^18^ for all primary tumors and CR cultures. Briefly, the MATH score is calculated as 100× MAD/median of the VAF, where MAD is the Median Absolute Deviation and VAF is the Variant Allele Frequencies.

### Copy number variations

We used Infinium Omni2.5-8 v1.3 beadchip from Illumina (San Diego, USA) according to the manufacturer’s instructions. DNA used for this assay was prepared from frozen primary tumor tissue material from G2202 specimen sample and normal and tumor CR cells using Qiagen’s DNeasy blood and tissue kit. Data was collected at the Yale University’s Keck Biotechnology Resource Laboratory and analyzed by Genomic Services at Yale University by Dr. Xiting Yan to generate PennCNV plots for each chromosome.

## Electronic supplementary material

Supplemental Information
