# Supplementary material for: Patient-derived conditionally reprogrammed cells maintain intra-tumor genetic heterogeneity
Source: Sci Rep. 2018 Mar 6;8:4097. doi: 10.1038/s41598-018-22427-1 (PMC5840339; doi:10.1038/s41598-018-22427-1)
Supplement: Supplementary file 1 — Supplemental Information [file 41598_2018_22427_MOESM1_ESM.pdf]

Supplemental Information

Patient-derived conditionally reprogrammed cells maintain intra-tumor genetic heterogeneity

Bruna R. S. Correa[1*§], Joanna Hu[2*], Luiz O. F. Penalva[3], Richard Schlegel[4], David L. Rimm[2], Pedro A. F. Galante[1≠], Seema Agarwal[2,4≠]

[1]Centro de Oncologia Molecular - Hospital Sírio-Libanês, São Paulo-SP. 01308-060, Brazil

[2]Department of Pathology, Yale University, New Haven, CT 06510, USA

[3]Children's Cancer Research Institute – UTHSCSA, San Antonio, TX 78229, USA

[4]Department of Pathology, Center for Cell Reprogramming, Georgetown University Medical Center, Washington, DC 20007, USA

[§]Present address: Centre for Genomic Regulation (CRG), Barcelona 08003, Spain

[*≠]Authors contributed equally to this work

[*]Corresponding authors:
Pedro A. F. Galante
pgalante@mochsl.org.br
Seema Agarwal
sa1137@georgetown.edu

Key words: Heterogeneity, lung cancer, conditional reprogramming

Supplemental Method:

Cells were grown on coverslips, fixed with 4% freshly prepared Formaldehyde solution, permeabilized with 0.2% Triton X-100, blocked with 2% Bovine serum albumins in PBS buffer and incubated with pan-cytokeratin antibody (Dako, clone AE1/AE3) diluted in the block for an hour at room temperature (1:100 dilution) followed by an hour incubation with anti-mouse HRP (Jackson Immuno Research Laboratories, USA). Signal was developed for 5 min in 3,3′-diaminobenzidine solution (Dako; prepared according to manufacturer instructions), followed by counterstaining for 1 minute with hematoxylin (Tacha’s automated hematoxylin, BioCare Medical, Concord, CA). Slides were mounted with Prolong Gold mounting medium (Life Technologies). For H&E staining, coverslip was stained with Hematoxylin solution for a minute, rinsed well in water followed by 95% ethanol for 30 seconds. Counterstaining of coverslip was done with 2 min incubation with Eosin Y solution followed by dehydration of slide and mounting with resinous mounting medium (Thermo Fisher, USA). Chromogenic stained slides and H&E slides were scanned to create bright-field digital images using the ScanScope CS (Aperio, Vista, CA) at 40x magnification. All digital images were viewed in ImageScope.

Supplementary Figure 1S: Stable conditionally reprogrammed cell cultures maintained the morphology of tumor of origin. A. Examples of adenocarcinoma (ADCA) and squamous cell carcinoma (SCC) conditionally reprogrammed (CR) cell morphology from two individual patients. H&E and pan-cytokeratin chromogenic staining

show the preservation of cell morphologies. Arrowheads indicate the desmosome structure at the cell junctions in SCC.

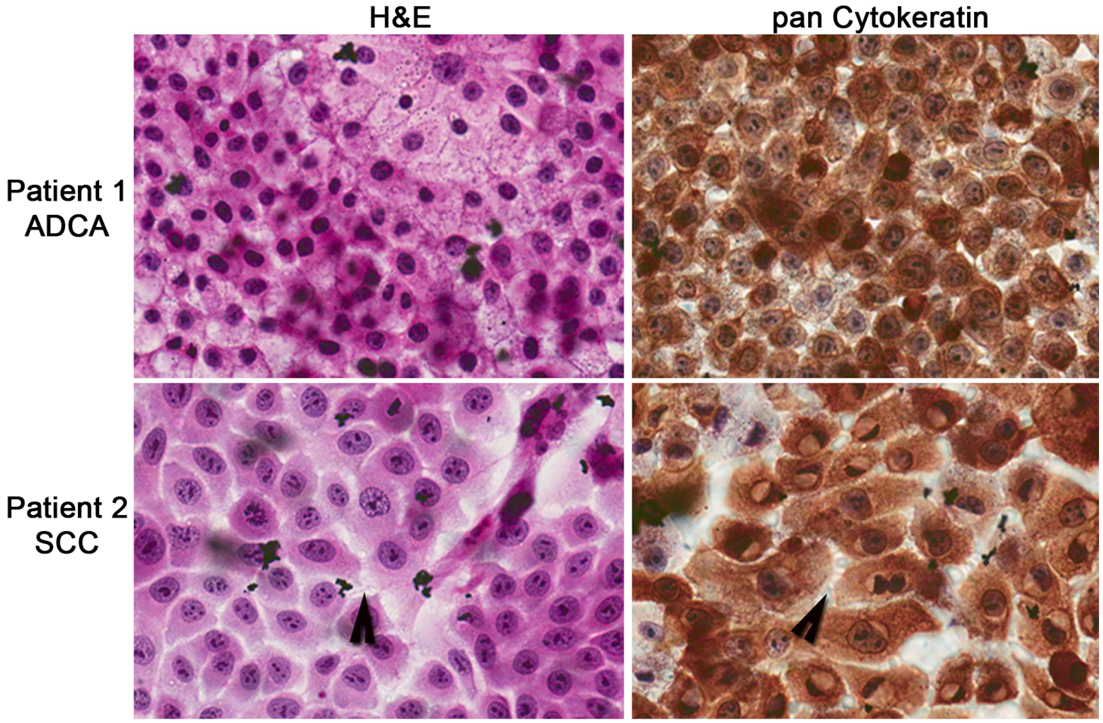

Supplemental Figure 2S. CNV profiles shown as high-resolution PennCNV plots for all chromosomes for G2202 sample. F is frozen primary tissue sample; N is adjacent normal conditionally reprogrammed (CR) cells and C is tumor CR cells.

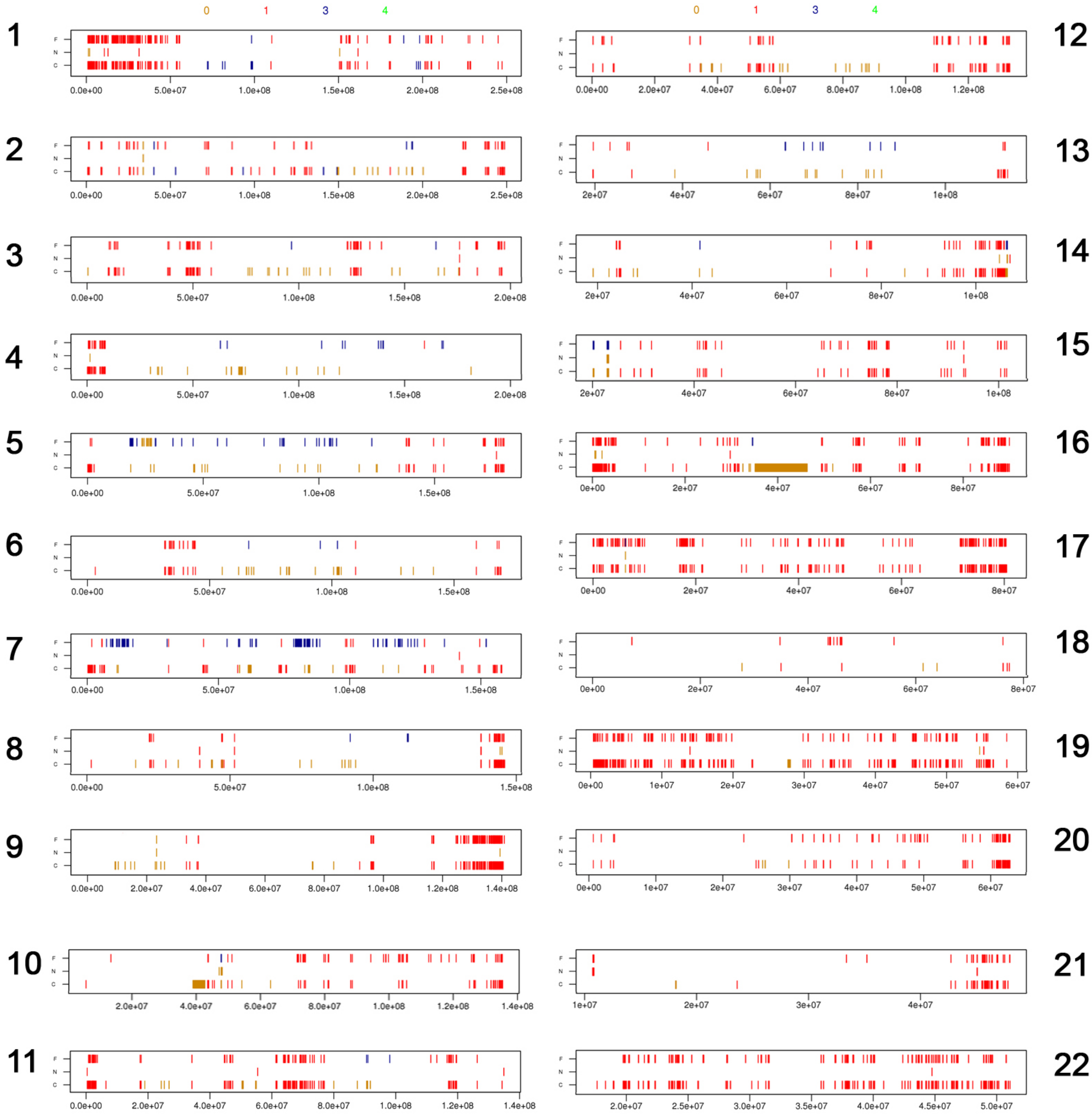

Color coding: 0= homozygous deletion; 1= heterozygous deletion; 3&4= amplification
